# Switch to once or twice daily unboosted atazanavir in a cohort of stable HIV patients: strong differences in drug exposure and virological outcome

**DOI:** 10.1186/1758-2652-13-S4-P47

**Published:** 2010-11-08

**Authors:** T Baudry, A Boibieux, MC Gagnieu, L Cotte, JM Livrozet, P Miahles, D Makhloufi, C Chidiac, D Peyramond, T Ferry

**Affiliations:** 1 Hopital de la Croix Rousse, Service de Maladies Infectieuses et Tropicales, Lyon, France; 2 Hopital Edouard Herriot, Laboratoire de Pharmacologie, Lyon, France; 3 Hotel Dieu, Service d'hépato-gastro-entérologie, Lyon, France; 4 Hopital Edouard Herriot, Service d'immunologie clinique, Lyon, France

## Purpose of the study

Switch to once daily unboosted (u) atazanavir (ATV) is an attractive option for HIV-infected patients with undetectable viral load, due to its convenience and favorable metabolic profile. However, due to a large interindividual variability, increased risk of inadequate ATV plasma concentrations (ATVc) and virological failure (VF) may be observed. Dividing the daily dose in a twice a day (BID) regimen could increase ATVc and improve virological success.

## Methods

In a prospective observational cohort of HIV-infected patients, all individuals with undetectable viral load who were switched to uATV during at least one month were retrospectively selected. ATVc were measured by a validated HPLC in all patients at least two weeks after the initiation of ATV. ATVc was considered inadequate when trough concentration was below 0,150 mg/L. Patients with once (400 mg QD) versus twice daily (200 mg BID) uATV were compared.

## Summary of results

From 2002 to 2009, 58 patients who received a total of 69 uATV based-regimens (27 QD and 42 BID regimens) were included. At the start of uATV, patients received a median duration of 9 years (IQR 4-11) of antiretroviral therapy. The mean exposure time of uATV was 16 months. Clinical characteristics and comedications (including tenofovir) were similar in the two groups. ATVc was inadequate in 17 (63%) patients in the QD group versus 4 (9%) patients in the BID group (p<0.001). During the follow up, VF happened significantly more frequently in the QD group than in the BID group (6 [22%] versus 1 [2%]; p=0.012). VF or low ATVc lead to treatment discontinuation in10 (37%) QD regimens versus 4 (9%) BID regimens (p=0,06). No other significant differences were detected in the two groups.

## Conclusions

Despite possible bias due to the observational study design, strong differences were detected in plasma drug exposure and virological outcome in our study. When a switch to uATV is proposed in HIV-controlled patients, BID would be preferred to QD.

